# The importance of open emergency surgery in the treatment of acute mesenteric ischemia

**DOI:** 10.1186/s13017-015-0041-6

**Published:** 2015-09-26

**Authors:** Mansur Duran, E. Pohl, K. Grabitz, H. Schelzig, T. A. Sagban, F. Simon

**Affiliations:** Clinic for Vascular- and Endovascular Surgery, Heinrich-Heine-University, Moorenstr. 5, 40225 Düsseldorf, Germany

**Keywords:** Mesenteric ischemia, Mesenteric infarction, Superior mesenteric artery, Vascular reconstruction, Endovascular treatment

## Abstract

**Objective:**

Acute mesenteric ischemia (AMI) is a complex disease with a high mortality rate. A patient’s chance of survival depends on early diagnosis and rapid revascularization to prevent progression of intestinal gangrene. We reviewed our experience with open surgery treatment in 54 cases of AMI.

**Methods:**

A monocentric retrospective study was conducted between 01/01/2001 and 04/30/2014; 54 AMI patients with a mean age of 56.6 years underwent surgery (26 women and 28 men). Retrospectively, the risk factors, management until diagnosis, vascular therapy and follow-up were evaluated.

**Results:**

The symptom upon admission was an acute abdominal pain event. The delay time from admission to surgery was, on average, 13.9 h (*n* = 34). The therapeutic procedures were open surgical operations. The complication rate was (53.7 %) (*n* = 29). The 30-day mortality was 29.6 % (*n* = 16). The late mortality rate was 24.1 % (*n* = 13), and the cumulative survival risk was 44.6 %. Survival was, on average, 60.54 months; however, in the over 70-year-old patient subgroup, the survival rate was 9.5 months (*p* = 0.035). The mortality rate was 27 % (*n* = 22) in the <12 h delay group, 20 % (*n* = 5) in the 12–24 h delay group, and 50 % (*n* = 7) in the > 24 h delay group.

**Conclusions:**

The form of therapy depends on the intraoperative findings and the type of occlusion. Although the mortality rate has decreased in the last decade, in patients over 70 years of age, a significantly worse prognosis was seen.

## Introduction

Cardiac arrhythmia with embolism is a frequent cause of acute mesenteric ischemia (AMI). An acute closure of the superior mesenteric artery (SMA) frequently leads to irreversible damage of the intestinal mucosa within 6 h [1]. The status of the celiac and SMA - celiac collaterals as well as the hemodynamic status of the patient can be important for AMI. Patients primarily present with an acute pain event, which is followed by an interval of reduced pain intensity due to the decline of intramural pain receptors arising from the hypoperfusion of the intestinal wall. As a result, this disease is often not initially recognized as a vascular emergency, which leads to the possibility of delays in making the appropriate diagnosis and initiating treatment. Further along the course of the disease, intestinal wall gangrene develops due to the destruction of the mucosal barrier, followed by bacterial translocation, which may lead to peritonitis, sepsis and multi-organ failure. Therefore, AMI comes with a high mortality rate (50–70 %) [2]. Prognostic factors include delays in diagnosis and in the revascularization of the intestine, age of the patient and comorbidities [1]. AMI increases up to 10 % in patients older than 70 years with an acute abdomen [1]. Predisposing risk factors include heart failure, coronary heart disease (CHD), hypertension, peripheral arterial disease (PAD) and location of the occlusion.

The mortality rate is directly correlated with the delay interval, and in the case of rapid therapy, it is approximately 0–10 %. The mortality rate increases to 50–60 %, with a delay of 6–12 h, and then to 80–100 % in cases where the delay interval is greater than 24 h [3]. Peripheral vessel occlusion is associated with lower mortality than is central vessel occlusion because of the better collateralization [3]. Non-occlusive mesenteric ischemia (NOMI) represents a special form of vessel occlusion that is caused by vasospasms of the mesenteric arterial vessels. This form is more difficult to diagnosis than the occlusive form and may therefore entail further delays.

The aim of this retrospective study is to describe AMI as a vascular emergency in an effort to sensitize primary clinicians in all disciplines. The study illustrates the evaluation of risk factors, management until diagnosis, vascular therapy and outcome.

## Methods

In a retrospective study, 54 patients with AMI between 01/01/2001 and 04/30/2014 are presented. Patients who developed AMI as a complication of other operations or interventions were excluded from the study.

Mortality, morbidity, risk factors, management until diagnosis, vascular therapy and outcome were retrospectively evaluated. Institutional Review Board approval was waived because of the retrospective nature of the study. All patients gave their written and informed consent prior to surgery.

The patient characteristics (risk factors, cause of occlusion, operational procedure, complications) are summarized in Tables 1, 2 and 3.

**Table 1:** Risk factor- dependent analysis of survival was performed in a logistic regression

Variable	*n* = 54 (%)	*P* value
Age (mean ± SD)	56.61 ± 16.21	0.035
Gender	54 (100)	0.451
Arterial hypertension	31(57.4)	0.172
Coronary heart disease	15 (27.8)	0.959
Diabetes mellitus	11 (20.4)	0.567
Hyperlipidemia	10 (18.5)	0.564
Peripheral vascular disease	8 (14.8)	0.036
Smoking history	11 (20.4)	0.222
Arrhythmias	8 (14.8)	0.480
Second look	23 (42.6)	0.121

The level of significance was defined at *p* <0.05

*SD* Standard deviance

**Table 2:** Operational procedure and cause of occlusion of patients (*n* = 54) undergoing open surgical therapy

Parameter	*n* (%)
Operational procedure	
TEA	22 (40.7)
Thrombectomy/embolectomy	14 (25.9)
Bypass	13 (24.1)
Resection of the dissection membrane	11 (20.3)
SMA-transposition	7 (13.0)
Visceral surgery	8 (14.8)
Explorative laparotomy	3 (5.6)
Cause of occlusion	
Arterial embolism	16 (29.6)
Arteriosclerosis combined with local thrombosis	13 (24.0)
Arterial thrombosis	12 (22.2)
Arterial dissection	9 (16.7)
Local dissection	2 (3.7)
Unclear cause of occlusion	2 (3.7)

**Table 3:** Complications after surgery

Parameter	*n* (%)
Vascular occlusion	8 (14.8)
Ischemia of the gut	8 (14.8)
Acute kidney failure	6 (11.1)
Multi-organ failure	5 (9.2)
Wound healing disorder	5 (9.2)
Arrhythmias	3 (5.6)
Septic shock, respiratory failure, abdominal compartment syndrome, rebleeding; each *n* = 2	8 (14.8)
Peritonitis, ileus, Leriche syndrome, Non occlusive disease, liver failure, delirium, cholecystitis; each *n* = 1	7 (13.0)

### Statistical analysis

Statistical analysis was performed in a logistic regression for risk factors. The relationships between bypass, delay interval and survival were assessed using the Fisher, Kruskal-Wallis- and *χ*^2^-tests, as well as by cross tables. Survival curves and survival distributions were determined using the Kaplan-Meier method and the log-rank test. The level of significance was defined at p <0.05. The SPSS statistical package (IBM Corp. Released 2013. IBM SPSS Statistics for Windows, Version 22.0. Armonk, NY: IBM Corp.) was used for all statistical analyses.

## Results

Patients presented at the clinic with an acute pain event of the abdomen. The average age was 56.6 years (11–85 years, of which 12 patients (22.2 %) were over 70 years old). The youngest patient (11 years old) had cardiac arrhythmia with embolism and occlusion of the abdominal aorta and SMA.

Diagnosis was provided by CT angiography with contrast medium (83.3 %, *n* = 45), conventional angiography (53.7 %, *n* = 29), duplex sonography (27.8 %, *n* = 15) or magnetic resonance imaging (MRI) (1.9 %, *n* = 1). Altogether, a total of 45 out of 54 patients (83.3 %) had leukocytosis (>10 000 /μl), 48 out of 52 patients (92.3 %) had an increase of C-reactive protein (>0.5 mg/dl) and 18 out of 26 patients (69.2 %) had an increase of L-lactate (>1.6 mmol/l). The delay from admission to surgery was, on average, 13.9 h (*n* = 34, 63 %). In 20 patients, the delay could not be determined. Logistical regression revealed that PAD (*p* = 0.036) and age (*p* = 0.035) were the factors with the greatest influence on survival (Table 1). The 30-day mortality was 30.8 % (*n* = 16), the late mortality was 25 % (*n* = 13) and the overall mortality was 53.7 % (*n* = 29). For the <12 h delay group, the mortality was 27.2 % (*n* = 22), and the mortality in the group with 12–24 h delay was 20 % (*n* = 5) and was 57.1 % (*n* = 7) in the > 24 h after admission group. The delay interval was known in 34 cases. Statistical analysis of the delay interval using the crosstab test and the *χ*^2^ test did not reveal statistical significance on survival (*p* = 0.42).

**Fig. 1:**
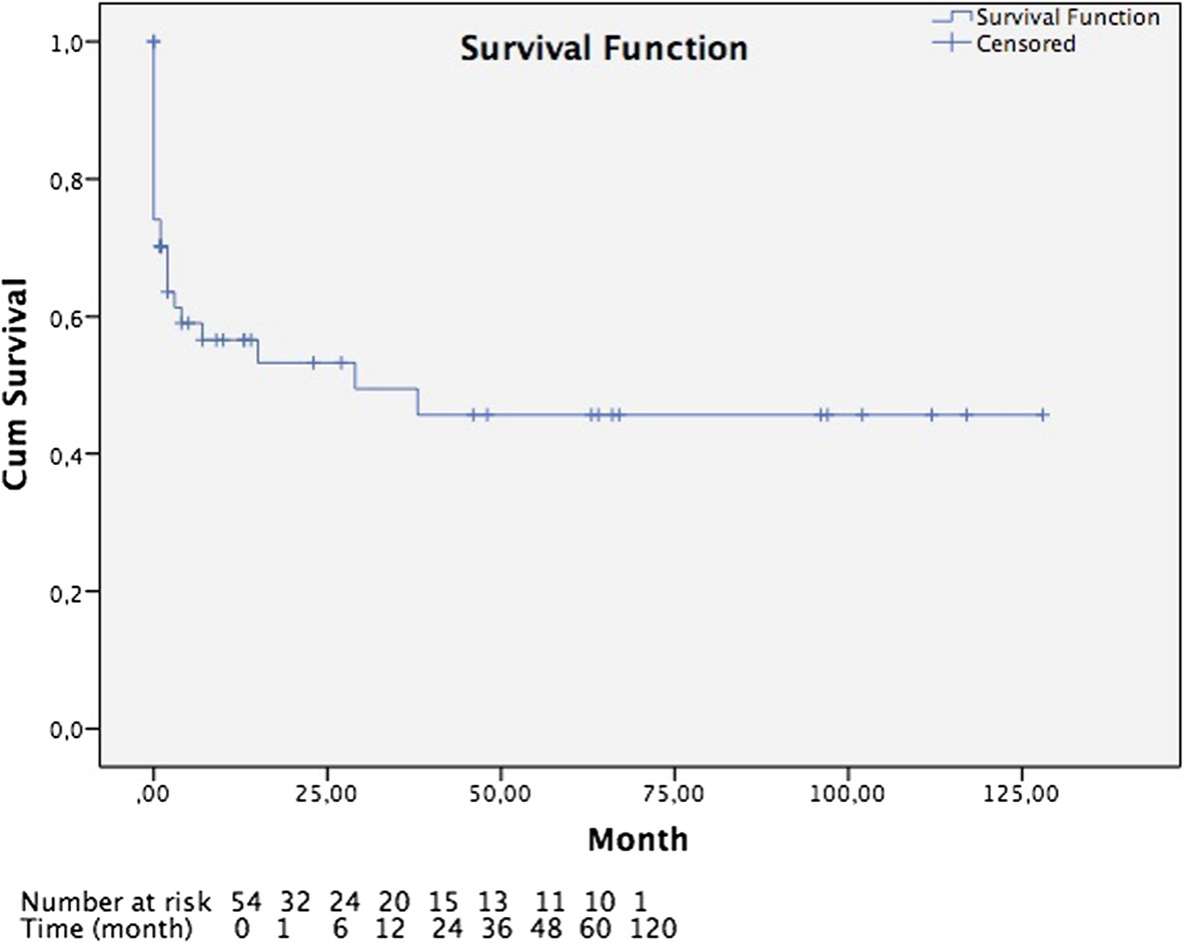
Survival status of 54 patients with open surgical therapy

The cumulative survival risk was 44.6 %, with an average survival of 60.5 months (men: 70.2 months, women: 42.8 months) (Fig. 1). The log-rank test did not show a significant effect of gender on survival (*p* = 0.529).

**Fig. 2:**
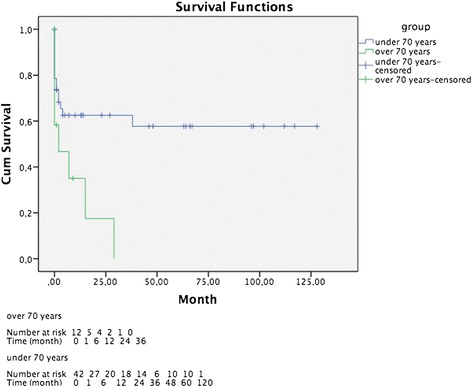
Survival status oft he groups under and over 70 years of age

The under-70-year-old patient group had a survival-time of 72.6 months, in contrast to the over 70 age group, which only had a survival-time of 9.5 months. Statistical analysis (log rank test) showed a significant difference between the age groups (*p* = 0.035) (Fig. 2).

The causes of death during the early phase (<30 days) included necrosis of the gut (*n* = 6), multi-organ failure (*n* = 5), and other causes (*n* = 5). In the late phase (>30 days), the causes of death included ischemia of the gut (*n* = 2), other causes (*n* = 3), and unclear cause of death (*n* = 8).

The primary patency rates were 80 % for transposition, 62 % for bypass, 53 % for TEA, and the secondary rates were 77 % for grafts and 79 % for TEA. Overall, there was no significant relationship (cross-tab, Fisher test, *χ*^2^ test and Kruskal-Wallis test) between the choice of the bypass (“French bypass” or other bypass) and the choice of the material (vein or alloplastic material) used in terms of survival.

Twenty-five patients survived the AMI, whereas 7 patients could not be located for follow-up and 3 patients refused the investigation.

The long-term results are based on 15 patients in the follow-up (4 months - 9 years). During follow-up, one patient complained about diarrhea and another patient suffered from nausea and vomiting. A total of 13 patients (86.7 %) were asymptomatic and stated a positive quality of life.

## Discussion

The mortality in patients with AMI remains high despite the diagnostic, surgical and endovascular developments in recent decades. There was a decline in mortality rate from 80–100 % in the 1970s to 50–70 % in the last decade. The decrease has been attributed to the better management of diagnosis and therapy [4]. The cause of visceral ischemia is an important factor. The mortality rate after surgical therapy for arterial embolism (54.1 %) and venous thrombosis (32.1 %) in a meta-analysis of Schoots et al. [5] was very high. This number rose after surgical treatment for arterial thrombosis (77.4 %) and NOMI (72.7 %). In our study, the 30-day mortality rate was 30.8 %. Decisive factors that contribute to the severity of the disease are a late diagnosis, the resulting delay in vascular therapy and the pre-existing co-morbidities of many patients. An early diagnosis, prior to the development of bowel necrosis with peritonitis, is one of the most important prognostic factors [6]. Statistical analysis of the delay interval did not reveal statistical significance on survival in our study because of the low number of cases (*n* = 34).

CT scanning with contrast agents is the current gold standard in instrument-based diagnostic testing, providing both high sensitivity and specificity. In contrast and despite its high sensitivity and specificity, MRI requires further development because of the longer duration of the investigation and its low availability. It therefore plays only a minor role in the diagnosis of peripheral embolisms of the SMA, NOD and of acute mesenteric ischemia [7, 8].

In cases of suspected AMI, the emergency diagnosis should include biphasic contrast computed tomography (CECT) with multi-planar reconstruction at 3 levels. The venous phase of the CT is necessary for the diagnosis of mesenteric vein thrombosis [9]. In our study, CT with contrast agents was the gold standard in instrument-based diagnostic testing, having replaced conventional angiography as our imaging modality of choice.

Non-specific serum lactate is often used as a diagnostic parameter. An elevated lactate level (>2.2 mmol/l) reflects the late phase of AMI with a transmural bowel infarction, where release of lactate into the bloodstream is caused by anaerobic metabolism with bacterial translocation. Serum lactate, however, cannot detect the early phase where there is only damage to the intestinal mucosa [10, 11]. Another serum parameter that can be used for diagnosis is the D-dimer, which, although sensitive to the early phase, has very low specificity [4].

As future biomarkers for the early phase of AMI, intestinal fatty acid-binding protein (I-FABP), α-glutathione S-transferase (GST) and D-lactate may play an important role. I-FABP and GST are localized in the small intestinal mucosa, whereas D-lactate is a natural degradation product of intestinal bacteria. These markers may appear early in the bloodstream if damage to the mucosa of the small intestine occurs, leading to a loss of enterocytes as the first sign of ischemia. However, the reliability of these serum markers has yet to be demonstrated in randomized controlled prospective studies [12]. In our daily clinical practice we used only L-lactate, leukocytosis and C-reactive protein as parameters for AMI. Biochemical markers, such as I-FABP, GST and D-lactate, must be further studied to determine their roles as valid biomarkers for AMI, and therefore, they cannot be used in daily clinical practice [11, 12]. L-lactate, currently the most commonly used marker, is not specific enough, however, and is only present during the late phase of AMI [13].

In a thrombectomy or embolectomy, the thrombus is recovered through a transaortic or transmesenteric arteriotomy. The arterial occlusion is closed by either direct suture or vein patch depending on the vessel diameter. As atherosclerotic plaques cause occlusion of the visceral arteries, these plaques can be removed either indirectly transaortally or directly via an open endarterectomy (TEA). Surgical access can be performed by a laparotomy or through a thoracoabdominal approach [14]. An SMA transposition can be used for short segment stenosis and occlusions of the SMA. Here, the artery is discontinued and reinserted further distally into the infrarenal aorta [15].

On the other hand, in cases of stenoses extending to a large portion of the vessel wall, the bypass procedure is the method of choice. The great saphenous vein or an alloplastic material (PTFE, Dacron) are available as bypass materials that may be applied antegrade or retrograde. In the retrograde procedure, where the origin of the bypass is located distal to the SMA, the vessel substitutes should be guided behind the left renal vessels. The renal vessels serve as a fulcrum to prevent kinking of the bypass. Due to its complex correct anatomic description, this bypass is called the “French bypass”. The “French bypass” combines the advantages of ante- and retrograde visceral bypasses. The procedure allows distal segments of the SMA to be reconstructed [16–18]. If the lumen of the SMA is occluded due to a dissection membrane that separates the true from the false lumen, the membrane can be resected transaortally following direct suturing of the aorta. In our center, we used all of these surgical procedures (Table 1) and saw the best results for transposition, with a primary patency rate of 80 %. Overall, there was no significant relationship between the choice of the bypass and the choice of the material used in terms of survival. The results from the first case series of 64 patients (1979–2000) were published in 2002 [19]. Compared to our 2002 study [19], no difference in terms of age, gender, risk profile or cause of occlusion could be found in the present work. However, the new study showed a reduction in the overall mortality rate, from 67 to 30 %, due to the improved management of the diagnostic and treatment options.

The modern treatment of AMI should primarily be performed by revascularization of the intestine.

A visceral surgical operation with bowel resection of necrotic parts can be performed afterwards. In modern vascular surgery, both endovascular and open surgical treatment options should be considered.

Preoperatively, both clinical and CT morphological aspects must be considered, whether the patient presents with peritonitis or an embolic or thrombotic occlusion of the SMA. The endovascular treatment option is recommended only in patients without peritonitis [1].

In the case of an AMI in combination with peritonitis, exploratory laparotomy with vascular therapy is the method of choice.

Five non-randomized studies have reported a comparison between open and endovascular surgery. In a retrospective study conducted within a single center, no difference in mortality between the two methods could be shown [4]. However, another study reported lower morbidity and mortality using the endovascular method [20]. Three other multicenter studies were national supra-regional studies: lower rates of bowel resection and lower mortality and morbidity were seen in these studies for the endovascular method [21, 22]. Therefore, if endovascular therapy appears to be possible, this method should be carried out whenever possible after the physician has excluded peritonitis.

Open surgery can also be used in patients with peritonitis in the late phase of AMI.

The poorer outcomes compared to endovascular procedures are due to the poorer conditions of a late phase AMI.

Our study is, however, limited by its retrospective nature and as a single center study in general validity.

## Conclusions

AMI is an important differential diagnosis in acute abdomen. The diagnosis should be made by CT angiography without a delay interval. The type of therapy depends on the intraoperative findings and the type of occlusion. In patients over 70 years of age, a significantly worse prognosis was seen.
